# Update der Point-of-care-basierten Gerinnungstherapie: Systeme, Reagenzien, gerätespezifische Behandlungsalgorithmen

**DOI:** 10.1007/s00101-023-01368-z

**Published:** 2024-01-23

**Authors:** Felix C. F. Schmitt, Herbert Schöchl, Kathrin Brün, Sascha Kreuer, Sven Schneider, Stefan Hofer, Christian F. Weber

**Affiliations:** 1 Klinik für Anästhesiologie, Universitätsklinikum Heidelberg, Im Neuenheimer Feld 420, 69120 Heidelberg, Deutschland; 2 Ludwig Boltzmann Institut für Traumatologie, AUVA Research Center, Wien, Österreich; 3 Klinik für Anästhesiologie und Intensivmedizin, AUVA Unfallkrankenhaus, Salzburg, Österreich; 4 Klinik für Anästhesiologie, Intensivmedizin und Schmerztherapie, Universitätsklinikum des Saarlandes, Homburg, Deutschland; 5 Medizinische Fakultät, Universität des Saarlandes, Homburg, Deutschland; 6 Klinik für Anästhesiologie, Westpfalz-Klinikum Kaiserslautern, Kaiserslautern, Deutschland; 7 Klinik für Anästhesiologie, Intensiv- und Notfallmedizin, Asklepios Klinik Wandsbek, Hamburg, Deutschland; 8 Klinik für Anästhesiologie, Intensivmedizin und Schmerztherapie, Universitätsklinikum Frankfurt, Frankfurt am Main, Deutschland

**Keywords:** Trauma, Blutung, Koagulopathie, Hämorrhagie, Massiv-Transfuion, Trauma, Bleeding, Coagulopathie, Hemorrhage, Massiv-transfusion

## Abstract

Point-of-care(POC)-taugliche viskoelastische Testverfahren (VET) sind im klinischen Einsatz weitverbreitet. Aufgrund des erweiterten Spektrums an verfügbaren Geräten und insbesondere durch die Entwicklung neuer Testansätze bzw. -methoden ist aus Sicht der Autoren ein Update der aktuellen Behandlungsalgorithmen notwendig. Ziel dieses Artikels ist es, einen Überblick sowohl über die aktuell verfügbaren VET-Geräte als auch über die dazugehörigen Reagenzien zu vermitteln. Darüber hinaus werden 2 Therapiealgorithmen für die am häufigsten im deutschsprachigen Raum eingesetzten VET-Geräte vorgestellt.

**Zusatzmaterial online:**

Die Online-Version dieses Beitrags (10.1007/s00101-023-01368-z) enthält die Algorithmen „POC Algorithmus_ClotPro“ und „POC Algorithmus_ROTEM delta“ für Sie als Download-Datei. Bitte scannen Sie dafür den QR-Code oder gehen Sie auf www.springermedizin.de und geben Sie den Titels des Artikels ein.

## Abstract (en)

Viscoelastic test (VET) procedures suitable for point-of-care (POC) testing are in widespread clinical use. Due to the expanded range of available devices and in particular due to the development of new test approaches and methods, the authors believe that an update of the current treatment algorithms is necessary. The aim of this article is to provide an overview of the currently available VET devices and the associated reagents. In addition, two treatment algorithms for the VET devices most commonly used in German-speaking countries are presented.

## Hintergrund

Perioperative Blutungen sind mit einer hohen Morbidität und Mortalität assoziiert [1]. Koagulopathien im Sinne einer insuffizienten Hämostase können vielfältige Ursachen haben. Eine relevant verringerte Konzentration von Gerinnungsfaktoren oder deren eingeschränkte Funktion, ebenso wie z. B. Thrombozytopenien und -pathien können mit einer erheblichen Blutungsneigung einhergehen [2–4]. Insbesondere ältere Patient:innen mit vorbestehenden Komorbiditäten und einer dauerhaften Einnahme von antithrombotischen bzw. antithrombozytären Präparaten haben ein höheres Risiko für substanzielle Blutverluste [5–7].

Ein Mangel an Gerinnungsfaktoren ist in der Regel das Resultat größerer Blutverluste, dem Verbrauch von Gerinnungsproteinen, insbesondere von Fibrinogen, und Dilutionseffekten [8]. Jede schwere, anhaltende Blutung führt durch diese Mechanismen früher oder später auch zu einer Koagulopathie. Somit kommt sowohl der frühen chirurgischen/interventionellen Blutstillung als auch der Optimierung des hämostatischen Potenzials eine zentrale Bedeutung im Management schwerer Blutungen zu.

Obwohl sich Standardgerinnungstests (SGT), wie die Prothrombinzeit (PTZ/Quick-Wert, INR) oder die aktivierte partielle Thromboplastinzeit (aPTT) zur Diagnose von perioperativen Blutungsereignissen als wenig hilfreich erwiesen haben und eigentlich nur zur Kontrolle verschiedener antithrombotischer Therapien entworfen und validiert wurden, werden sie dennoch häufig für diese Fragestellung herangezogen [9].

Point-of-care(POC)-taugliche viskoelastische Testverfahren (VET) ermöglichen eine differenzierte Diagnostik der zugrunde liegenden Gerinnungsstörung und werden daher auch von zahlreichen aktuellen Leitlinien explizit empfohlen ([10–17]; Tab. 1).

**Table 1:** 

Guidelines	Journal/Links
The European guideline on management of major bleeding and coagulopathy following trauma: sixth edition	Crit Care 2023 [10]
Management of severe peri-operative bleeding: Guidelines from the European Society of Anaesthesiology and Intensive Care: Second update 2022	EJA 2023 [11]
S3-Leitlinie Polytrauma/Schwerverletzten-Behandlung (AWMF Registernummer 187-023), Version 4.0 (31.12.2022)	AWMF 2022 [18]
Management of postpartum hemorrhage (PPH): algorithm of the interdisciplinary D‑A-CH consensus group PPH (Germany – Austria – Switzerland)	Anaesthesist 2014 [19]
Peripartum hemorrhage, diagnostics and treatment: update of the S2k guidelines AWMF 015/063 from August 2022	Anaesthesiologie 2022 [16]
2017 EACTS/EACTA Guidelines on patient blood management for adult cardiac surgery	J Cardiothorac Vasc Anesth 2018 [15]
Guidelines for Perioperative Care for Liver Transplantation: Enhanced Recovery After Surgery (ERAS) Recommendations	Transplantation 2022 [13]
Querschnitts-Leitlinien zur Therapie mit Blutkomponenten und Plasmaderivaten, Gesamtnovelle 2020	Bundesärztekammer 2020 [20]
Transfusion strategies in bleeding critically ill adults: a clinical practice guideline from the European Society of Intensive Care Medicine	Intensive Care Med 2021 [12]
Haematological management of major haemorrhage: a British Society for Haematology Guideline	Br J Haematol 2022 [17]

Standardgerinnungstests sind zur Diagnosestellung von perioperativen Blutungen wenig hilfreich

Verglichen mit SGT sind die Ergebnisse von VET wesentlich rascher verfügbar, insbesondere durch deren bettseitige Verfügbarkeit und können als Grundlage für eine gezielte Hämotherapie herangezogen werden [21]. Durch die Etablierung von Therapiealgorithmen, die auf einer VET-Diagnostik basieren, konnten zumeist eine Reduktion des Transfusionsbedarfs und in einigen Studien auch Überlebensvorteile nachgewiesen werden [22–26]. In den letzten Jahren wurde eine Reihe neuer VET-Geräte auf den Markt gebracht. Sie unterscheiden sich nicht nur technologisch voneinander, sondern auch in den verfügbaren Reagenzien und damit auch in ihrem diagnostischen Spektrum [27–29].

Im Unterschied zu den VET-Verfahren gibt es bislang keinen akzeptierten „Goldstandard“ für eine POC-taugliche Plättchenfunktionsdiagnostik. Daneben ist der Stellenwert der Plättchenfunktionstestung als zusätzliche Möglichkeit zur Detektion von Koagulopathien durch Studien wenig gesichert [30, 31].

Aufgrund des erweiterten Spektrums an verfügbaren VET-Geräten und insbesondere durch die Entwicklung neuer Testansätze bzw. -methoden ist aus Sicht der Autoren ein Update des vor über 10 Jahren vorgestellten Algorithmus, der von Weber et al. in dieser Zeitschrift publiziert wurde, erforderlich [32]. Ziel dieses Artikels ist es, einen Überblick sowohl über die aktuell verfügbaren VET-Geräte als auch über die dazugehörigen Reagenzien zu vermitteln. Darüber hinaus werden 2 Therapiealgorithmen für die am häufigsten im deutschsprachigen Raum eingesetzten VET-Geräte vorgestellt: (1.) basierend auf dem bereits 2013 publizierten POC-Algorithmus ein Update für das ROTEM® Delta und ein weiterer (2.) für das seit 2019 verfügbare ClotPro® (Abb. 2).

## Rückblick auf 25 Jahre Point-of-care-basierte Hämotherapie

Das erste VET-Gerät wurde schon 1948 von Hellmut Hartert entwickelt [33]. Dieses hämostaseologische Testverfahren hatte über die folgenden Jahrzehnte immer wieder Verwendung in unterschiedlichen klinischen Fragestellungen gefunden. Die ersten kommerziell verfügbaren VET-Geräte wurden zwar als POC-taugliche Methoden entwickelt, hatten aber einige gravierende Nachteile, die zu Beginn einem verbreiteten Einsatz oftmals im Wege standen. Das korrekte Pipettieren, insbesondere mit den anfangs verfügbaren Flüssigreagenzien, war fehleranfällig und somit die Reproduzierbarkeit der Testergebnisse bisweilen schwach [34]. Insbesondere im potenziell stressigen Umfeld, wie einer frühen Schwerverletztenversorgung oder im Rahmen kritischer peripartaler Blutungen, war die Akzeptanz dieser Methoden oftmals eingeschränkt.

Ein weiteres Manko der VET-Verfahren liegt in der eingeschränkten Standardisierung und limitierten methodenübergreifenden Übertragbarkeit der Testergebnisse. Eine Reihe von Anbietern hat mittlerweile Geräte mit durchaus unterschiedlichen Technologien auf den Markt gebracht [27, 29, 35, 36]. Die verfügbaren Reagenzien unterscheiden sich sowohl hinsichtlich der Aktivierungswege als auch der Zusammensetzung und der Konzentration z. T. erheblich. Daher dürfen Algorithmen nicht ohne Weiteres von einem Gerät auf das andere übertragen werden [27, 34].

Durch die Entwicklung und Einführung vollautomatischer, kartuschenbasierter Testsysteme bzw. solcher, die den Pipettierprozess signifikant vereinfachen, können Bedienungsfehler nunmehr deutlich reduziert werden [27].

## Wertigkeit von Standardgerinnungsbefunden

Häufig wird der Gerinnungsstatus im Rahmen akuter Blutungen durch die Bestimmung von SGT wie Prothrombinzeit (PTZ/Quick-Wert, INR) und aPTT erhoben. Bei der Messung von SGT werden allerdings nur pro- und keine antikoagulatorischen Aktivierungswege erfasst. Außerdem wird bei SGT nur die frühe Initiationsphase der Gerinnung abgebildet. Die Messung bricht bereits ab, wenn lediglich 5 % der gesamten Thrombinmenge gebildet wurden [37]. Die nach dem „zellbasierten Gerinnungsmodell“ folgende Amplifikations- und Propagationsphase bleibt dabei völlig unberücksichtigt [38]. Ebenso finden zelluläre Komponenten, wie Erythrozyten und Thrombozyten, die im Rahmen des Zentrifugationsvorgangs eliminiert werden, aber für eine suffiziente Hämostase unumgänglich sind, keine Berücksichtigung [38]. Darüber hinaus können z. B. auch ein Faktor-XII-Mangel oder Lupusantikörper die aPTT fälschlicherweise verlängern oder Fibrinspaltprodukte zu einer ungenauen Fibrinogenbestimmung führen. Deshalb ist es wenig verwunderlich, dass der prädiktive Wert von SGT zur Detektion einer Gerinnungsstörung oder Vorhersage einer Blutungsneigung nur gering ist [9, 39, 40].

## Viskoelastische Testverfahren

Viskoelastische Testmethoden (VET) ermöglichen im Unterschied zu SGT ein rasches und deutlich breiteres Spektrum des Gerinnungsablaufs, von der Initiierung der Gerinnung über die Geschwindigkeit der Gerinnselbildung bis hin zu Qualität, Stärke und Stabilität des gebildeten Clot [41]. Im Unterschied zu SGT erfolgt die Gerinnungsanalyse bei VET im Vollblut, somit kann der hämostaseologische Einfluss korpuskulärer Elemente (u. a. Thrombozyten, Erythrozyten) abgebildet werden. Die verfügbaren Geräte sind meistens POC-tauglich und Testergebnisse üblicherweise bereits nach wenigen Minuten verfügbar [21].

VET ermöglichen eine schnellere und umfassendere Übersicht des Gerinnungsablaufs

**Figure 1:**
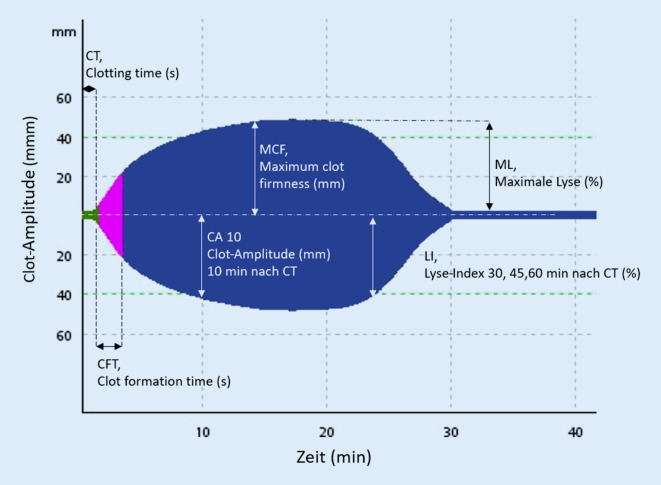

Wie schon erwähnt, unterscheiden sich diese Geräte technologisch z. T. erheblich voneinander. Bei TEG 5000®, ClotPro® (beide Fa. Haemonetics Corporation, Braintree, MA, USA) und ROTEM® (Fa. Werfen, Barcelona, Spanien) wird ein Cup mit Zitratblut gefüllt und entweder ein mit einem Torsionsdraht verbundener Pin (TEG 5000) oder ein zylindrischer Stempel (ROTEM® und ClotPro®) in die Blutprobe getaucht [41–43]. Je nach verwendeter Technologie rotiert entweder der Cup (TEG 5000® und ClotPro®) oder der Stempel kontinuierlich in einem Winkel von ca. 4,75° nach rechts und links. Die Bildung erster Fibrinfäden zwischen Stempel und Wand des Cup vermindert die Rotation des Cup oder Stempels in Abhängigkeit von der zunehmenden Gerinnselfestigkeit. Diese Einschränkung der Bewegung wird dann als eine Kurve über die Zeit grafisch dargestellt, und unterschiedliche gerinnungsrelevante Parameter können ausgelesen werden (Abb. 1).

Bei den VET-Geräten TEG 6s® wird die Blutprobe in eine Messkartusche eingebracht, vollautomatisch angesaugt und mit unterschiedlichen Aktivatoren versetzt [28, 35]. Das entstandene Gerinnsel wird mittels Schallwellen in Schwingung versetzt. Die zunehmende Gerinnselsteifigkeit vermindert die induzierte Vibration. Dieser Prozess kann ebenfalls als Kurve über die Zeit grafisch dargestellt werden.

## VET-Parameter

Die Clotting time (CT) repräsentiert die Zeit (s) bis zum Beginn der Gerinnung und dem Erreichen einer Gerinnselamplitude von 2 mm. Die Clot formation time (CFT) gibt die Zeit (s) vom Ende der CT (2 mm Amplitude) bis zu einer Gerinnselamplitude von 20 mm an. Die Gerinnselstärke 5 oder 10 min nach der CT wird als Clot-Amplitude (CA 5/10) bezeichnet. Die maximale Gerinnselfestigkeit („maximum clot firmness“, MCF) gibt die maximale Amplitude (in Millimetern) der Gerinnselstärke an. Der Lyse-Index nach 30, 45 und 60 min repräsentiert die Abnahme der Gerinnselfestigkeit ausgehend von der MCF in Prozent. Die maximale Lyse (ML) bildet die maximale Reduktion (in Prozent) der Clot-Amplitude ab.

## Verfügbare Reagenzien für VET

Verschiedene Aktivatoren und/oder Inhibitoren unterstützen die Differenzialdiagnose einer möglichen zugrunde liegenden Gerinnungsstörung. Dabei werden, ähnlich der Prothrombinzeit, extrinsische oder vergleichbar zur aPTT intrinsische Aktivierungswege genutzt. Tab. 2 fasst die momentan verfügbaren Assays mit den jeweiligen Aktivierungswegen für ROTEM® und ClotPro®, zusammen.

**Table 2:** 

Assay-Namen	Aktivatoren	Information
**Intrinsische Tests**		
INTEM (ROTEM®) IN-Test (ClotPro®)	Ellagsäure	Über Kontaktaktivierung wird die intrinsische Gerinnungskaskade gestartet. Sie liefert, ähnlich der aPTT, Informationen über die Faktoren XII, XI, IX, VIII und ist heparinsensitiv
*Extrinsische Test*		
EXTEM (ROTEM®) EX-TEST (ClotPro®)	„Tissue factor“	Vergleichbar zur PTZ liefern diese Tests Informationen über die extrinsische Gerinnungskaskade, insbesondere FVII. Wenig heparinsensitiv
*Fibrinpolymerisationstests*		
FIBTEM (ROTEM®)	Cytochalasin	Information über die Stärke des Fibringerinnsels. Ermöglicht die Differenzialdiagnose einer verminderten Clot-Amplitude in den Globaltests
FIB-Test (ClotPro®)	Cytochalasin + Tirofiban	
*Heparinase-Tests*		
HEPTEM (ROTEM®) HEP-Test (ClotPro®)	Intrinsisch aktivierter Test + Heparinase	Erlaubt den Ausschluss eines Heparineffekts oder einer Heparinrestwirkung. Bei Anwesenheit von Heparin ist die CT im Heparinasetest kürzer als die INTEM/-Int-Test-CT
*Lyse-Test*		
APTEM (ROTEM®) AP-Test (ClotPro®)	Extrinsisch aktivierter Test + Tranexamsäure	Gibt Aufschluss über den Effekt eines Antifibrinolytikums auf das Gerinnsel
t‑PA-Test (ClotPro®)	„Tissue plasminogen activator“	Liefert Information über eine potenzielle Fibrinolyse-Inhibierung („fibrinolytic shutdown“) z. B. bei schwerer Sepsis
**DOAC-spezifische Tests**		
ECA Test (ClotPro®)	Ecarinbasierter Test	Ecarin konvertiert Prothrombin zu Meizothrombin. Meizothrombin wird durch Thrombinhemmer inhibiert und führt zu einer Verlängerung der ECA-Test CT
RVV-Test (ClotPro®)	Russel’s Viper Venom Test	RVV aktiviert FX. Aktivierter FX wird durch Xa-Inhibitoren gehemmt ,und damit verlängert sich die RVV-Test-CT

Obwohl die Messprinzipien der verfügbaren Systeme vergleichbar sind, ergeben sich aufgrund von Unterschieden in der technischen Umsetzung der Geräte sowie der Verwendung unterschiedlicher Aktivatoren Abweichungen in den Messwerten und Referenzbereichen [44, 45]. Die Messergebnisse der Systeme sind somit nicht direkt miteinander vergleichbar, und Therapiealgorithmen, die für das jeweilige Gerät entwickelt wurden, können nicht einfach auf ein anderes Gerät übertragen werden.

## Einschränkung der VET-Methoden

VET-Methoden können bei Weitem nicht alle Aspekte einer Gerinnungsstörung abbilden. Ein Mangel an Von-Willebrand-Faktor (vWF) oder Faktor XIII (FXIII) kann mithilfe von VET nicht oder nur unzureichend detektiert werden. Plättchendysfunktionen werden mithilfe von VET ebenfalls nur ungenügend erfasst, nur das für die TEG entwickelte Platelet-Mapping™ (PM) ermöglicht die grobe Abschätzung einer möglichen Plättchenfunktionsstörung [46]. Auch DOAC können mit den üblicherweise verwendeten Standard-Assays nur mit unzureichender Sensitivität und Spezifität detektiert werden [47, 48], dafür sind DOAC-spezifische Reagenzien notwendig [29]. Die Geräte erwärmen, vergleichbar zu SGT im Labor, das zu analysierende Blut standardisiert auf 37 °C. Der Einfluss einer bestehenden Hypothermie auf die Gerinnung bleibt somit unerkannt. Weitere seltene, meist vorbekannte Koagulopathie-Ursachen, wie z. B. eine Hämophilie A/B, oder auch eine Leberfunktionsstörung können nicht oder nur sehr eingeschränkt detektiert werden [49].

## Plättchenfunktionstestung

Der Stellenwert der Plättchenfunktionstestung, als Ergänzung des diagnostischen Spektrums, im Rahmen schwerer Blutungen konnte bislang nicht eindeutig belegt werden [26, 30, 46]. Sowohl die technologische Umsetzung als auch die verfügbaren Reagenzien unterscheiden sich z. T. erheblich voneinander [50, 51].

Das Ausmaß einer bestehenden Plättchenhemmung muss mit speziellen Plättchenfunktionstests wie Multiplate®, Verifiynow®, PFA 100/200®, oder mithilfe des TEG-PM™ erhoben werden. Mithilfe unterschiedlicher Agonisten, wie Adenosindiphosphat (ADP), Kollagen, Arachidonsäure und Ristocetin werden verschiedene thrombozytäre Aktivierungswege stimuliert. Mit Ausnahme des Platelet Function Analyzer® (PFA) 100/200 kommen lediglich statische Messmethoden zur Anwendung. Die für die Plättchenaktivierung notwendigen Scherkräfte bleiben somit meist unberücksichtigt.

Bislang haben Therapie-Algorithmen, basierend auf einer Plättchenfunktionsdiagnostik, oftmals negative Resultate geliefert [31, 51]. Dies liegt einerseits daran, dass kein allgemein akzeptierter „POC-Goldstandard“ für die Plättchenfunktionsdiagnostik etabliert werden konnte und andererseits die momentan verfügbaren Geräte primär entwickelt wurden, um den Einfluss von Thrombozytenfunktionshemmern, wie Aspirin oder P2Y_12_-Antagonisten, zu erfassen und weniger, um eine potenzielle Blutungsursache durch eine Plättchendysfunktion zu detektieren [51]. Eine objektive Überlegenheit eines POC-Geräts gegenüber einem anderen konnte bislang nicht bestätigt werden. Die Vorhersagekraft aller Plättchenfunktionstests für den perioperativen Blutungs- und Transfusionsbedarf wird kontrovers diskutiert [30]. Daneben fanden sich signifikante Variabilitäten zwischen und innerhalb der verwendeten Tests [50]. Ein genereller und großzügiger Einsatz perioperativer Thrombozytenfunktionstestung muss somit kritisch gesehen werden [52].

Die kontroverse Studienlage spiegelt sich auch in den heterogenen Aussagen der aktuellen Leitlinien wider. „The European guideline on management of major bleeding and coagulopathy following trauma: 6th edition“ rät, im Unterschied zur 5. Auflage, nunmehr von der routinemäßigen Anwendung von POC-Plättchenfunktionstests ab [10]. Im Gegensatz dazu empfehlen die aktuellen S3-Polytrauma-Leitlinien von 2023 die Durchführung von Plättchenfunktionstests, jedoch ohne hinreichende Evidenz [18]. Die aktuellen ESAIC Guidelines sehen nur bei bestimmten Patientengruppen mit entsprechender Co-Medikation und positiver Blutungshistorie eine Indikation zur Plättchenfunktionstestung [11]. Aus diesem Grund wird auch in den hier vorgestellten Algorithmen die POC-Testung der Plättchenfunktion nur noch im Rahmen einer Ausschlussdiagnose empfohlen.

## Strukturierte Hämotherapie – Therapie-Algorithmus

## Optimierung der Rahmenbedingungen

### Acidose

Jede schwere Acidose führt sowohl zu einer relevanten Plättchenfunktionseinschränkung als auch zu einer deutlich reduzierten Gerinnungsfaktoraktivität. Bei Absinken des pH-Werts von 7,4 auf 7,1 vermindert sich die Thombinbildung um nahezu 50 % [53]. Des Weiteren scheint Fibrinogen im acidotischen Milieu einem beschleunigten Abbau zu unterliegen [54]. Eine alleinige Korrektur einer Acidose mittels Bikarbonat- oder Trispuffer führte aber zu keiner nennenswerten Verbesserung der Thrombingenerierung [55].

### Hypothermie

Ein Abfall der Körperkerntemperatur auf < 34 °C verursacht eine vermehrte Sequestration von Thrombozyten in Leber und Milz [56] und eine reversible Störung der thrombozytären Adhäsions- und Aggregationsfähigkeit [57, 58]. Das Temperaturoptimum von Gerinnungsenzymen liegt bei 37 °C. Daher führt eine Reduktion der Körperkerntemperatur auf < 34 °C zu einer verlangsamten Thrombingenerierung und damit auch zu einer verzögerten Gerinnselbildung [53]. Da die Gerinnungsdiagnostik sowohl bei SGT als auch VET-Verfahren meist standardisiert bei 37 °C durchgeführt wird, bleibt in der Regel der Einfluss einer Hypothermie auf den Gerinnungsprozess unentdeckt.

### Kalzium

Kalzium dient als Bindeglied zwischen den negativ geladenen Gerinnungsfaktoren und den ebenfalls negativ geladenen Phospholipiden und ist essenziell für die Plättchenaktivierung [59]. Da Kalzium bei zahlreichen Schritten der Gerinnungsaktivierung eine zentrale Rolle spielt, beeinträchtigt ein Mangel Gerinnungsabläufe maßgeblich. Insbesondere, wenn im Rahmen von Massivtransfusionen größere Volumina an zitrathaltigem Plasma transfundiert werden, können relevante Hypokalzämien rasch auftreten und bestehende Koagulopathien aggravieren [60]. Die notwendige Kalziumkonzentration für eine suffiziente Hämostase ist nicht exakt definiert, sollte jedoch > 0,9 mmol/l liegen [61]. Da 10 %ige Kalziumchloridlösung deutlich mehr ionisiertes Kalzium enthält als 10 %ige Kalziumgluconatlösung (270 mg/10 ml vs. 90 mg/10 ml) sollte es bei entsprechender Indikation bevorzugt eingesetzt werden [62].

### Hämoglobin

Eine ausreichende Masse an Erythrozyten ist nicht nur als Sauerstoffträger unabdingbar, sondern kann auch aufgrund rheologischer Effekte für die Blutgerinnung von Bedeutung sein. Erythrozyten drängen sowohl Plasma als auch Plättchen an den Randstrom von Gefäßen und steigern dadurch den Kontakt der Thrombozyten zu Gefäßläsionen [63]. Nach den aktuellen „European Trauma Bleeding-Guidelines“, der neuen Leitlinie „Management of severe perioperative bleeding“ und den Querschnitts-Leitlinien zur Therapie mit Blutkomponenten der Bundesärztekammer sollte in der akuten Hämorrhagie ein Hb-Wert von 7–9 g/dl angestrebt werden [10, 11].

## Antagonisierung von Antikoagulanzien und Plättchenhemmern

### Vitamin-K-Antagonisten

Vitamin-K-Antagonisten (VKA), wie Phenprocoumon oder Warfarin, hemmen die γ‑Carboxylierung der Vitamin-K-abhängigen Gerinnungsfaktoren FII, VII, FIX und FX. Der Marktanteil der VKA wurde in den letzten Jahren deutlich zurückgedrängt und beschränkt sich heute im Wesentlichen auf die Prophylaxe thromboembolischer Ereignisse bei valvulärem Vorhofflimmern, mechanischen Herzklappen sowie Patient:innen mit speziellen thrombotischen Risikofaktoren (z. B. Antiphospholipidsyndrom) [64].

### Diagnose von VKA

Die Therapie-Überwachung und Diagnose des Ausmaßes der Gerinnungshemmung mit VKA erfolgt idealerweise mittels INR. Von den VET-Verfahren würde sich, wenn überhaupt, nur ein extrinsisch aktivierter Test (EXTEM, EX-Test) eignen. Die INR ist allerdings wesentlich spezifischer und sensitiver und wurde auch explizit für diese Fragestellung entwickelt.

### Antagonisierung von VKA

Eine Reversierung von VKA ist zwar grundsätzlich mit Vitamin K möglich, die Neusynthese der Vitamin-K-abhängigen Gerinnungsfaktoren benötigt allerdings einige Stunden [65]. Im Rahmen akuter Blutungen kann mittels Prothrombinkomplexkonzentraten (PPSB) eine vollständige Reversierung von VKA zeitnah erreicht werden [66]. Die Konzentration der Gerinnungsfaktoren in PPSB ist etwa 25-mal höher als im Plasma. Verglichen mit FFP kann mit PPSB eine deutlich raschere und effektivere Antagonisierung des VKA-Effektes erreicht werden [67]. Die empfohlenen Dosierungen von PPSB zur Reversierung von VKA sind von der Ausgangs-INR abhängig und in Tab. 3 aufgelistet. Das thromboembolische-Risiko nach Gabe von PPSB wird mit ca. 5–6 % angegeben [68]. Bei einer bekannten Heparin-induzierten Thrombozytopenie darf nur heparinfreies PPSB verabreicht werden.

**Table 3:** 

INR	Dosis von PPSB
INR 2–4	25 U/kgKG
INR 4–6	35 U/kgKG
INR > 6	50 U/kgKG

### Direkte orale Antikoagulanzien (DOAC)

Im Gegensatz zu VKA nimmt der Marktanteil von DOAC in den letzten Jahren rasant zu. DOAC zeichnen sich im Vergleich zu VKA durch ein meist geringeres Blutungsrisiko bei vergleichbarer Effektivität und einer gut vorhersehbaren Pharmakokinetik und -dynamik aus [69]. Daher wird eine regelmäßige Bestimmung des individuellen DOAC-Spiegels nicht als notwendig erachtet, eine mögliche Kumulation sollte dennoch bei eingeschränkter Nieren- und Leberfunktion bedacht werden [70, 71].

#### Diagnostik von DOAC

##### Standardgerinnungstests.

SGT eignen sich nicht, um relevante Konzentration von DOAC nachzuweisen, da keine lineare Beziehung zwischen deren Plasmaspiegel und einer Verlängerung von PTZ, INR oder aPTT besteht [70].

##### Konzentrationsmessung von DOAC.

Dabigatrankonzentrationen im Plasma können mithilfe einer „verdünnten Thrombinzeit“ (Hemoclot-Tests®) oder mit der Ecarin clotting time (ECT) bestimmt werden [72]. Dabigatran bewirkt eine lineare, konzentrationsabhängige Verlängerung dieser Zeiten und ermöglicht damit ein Monitoring der antikoagulatorischen Aktivität. Chromogene Anti-Faktor-Xa-Aktivitäts-Tests können zur Einschätzung der Plasmaspiegel von Apixaban, Edoxaban und Rivaroxaban herangezogen werden, benötigen jedoch die Kalibrierung mit substanzspezifischen Reagenzien [70].

##### Viskoelastische Tests.

Augenblicklich stehen DOAC-spezifische VET nur für das ClotPro® zur Verfügung. Für Dabigatran wurde ein ecarinbasierter Test (ECA-Test) entwickelt, der eine ausgezeichnete Korrelationen von Dabigatran-Plasma-Spiegel und Clotting time aufweist. Für die Xa-Hemmer konnten mithilfe des Russel-Viper-Venom(RVV)-Tests klinisch relevante Cut-off-Werte definiert werden, die es erlauben, Konzentrationen > 50 ng/ml oder > 100 ng/ml, relativ sicher abzuschätzen (Tab. 4; [29]).

**Table 4:** 

DOAC-Plasma-Konzentration	ECA-Test, Clotting time	RVV-Test, Clotting time
> 50 ng/ml	> 190 s	> 170 s
> 100 ng/ml	> 315 s	> 190 s

#### Antagonisierung von DOAC

Zur Reversierung von DOAC stehen mittlerweile 2 spezifische Antagonisten zur Verfügung. [73, 74]. Idarucizumab, ein humanisiertes Antikörperfragment gegen Dabigatran, ist für lebensbedrohliche Blutungen zugelassen, kann aber auch vor dringlichen Operationen in einer Dosierung von 5 g eingesetzt werden [74]. Eine Antagonisierung von Dabigatran mit Idarucizumab normalisiert die ECA-Test-CT sofort und unmittelbar [75]. Auch die Dabigatran-Plasma-Spiegel sinken üblicherweise auf nichtmessbare Werte ab [76]. Nach Gabe von Idarucizumab ist zu beachten, dass bei sehr hohen initialen Dabigatranspiegeln ein „Rückshift“ von Dabigatran aus dem extravaskulären Bereich nach intravaskulär möglich ist; dieser kann zu einem erneuten kritischen Anstieg der Plasmakonzentration führen. In diesen Fällen sind eine wiederholte Spiegelbestimmungen erforderlich und evtl. auch eine repetitive Verabreichung von Idarucizumab notwendig [77, 78].

Andexanet alfa (AA) ist ein rekombinant hergestelltes, dem humanen Faktor Xa ähnliches Protein, das FXa-Inhibitoren mit hoher Affinität bindet [79]. Eine Zulassung von AA besteht momentan nur für lebensbedrohliche Blutungen unter Apixaban und Rivaroxaban, nicht jedoch für Edoxaban [73]. Bei Patienten unter Edoxaban kann alternativ ein Therapieversuch mit PPSB erwogen werden. Die aktuellen ESAIC Guidelines empfehlen auch für Rivaroxaban und Apixaban PPSB in einer initialen Dosierung von 25 IE/kgKG, auch wenn es hierfür aktuell keine Zulassung gibt [11].

Nach der Gabe von AA ist eine sichere Heparinisierung nicht mehr zuverlässig möglich, da AA auch antithrombinabhängige FXa-Hemmer, wie unfraktioniertes Heparin (UFH), inaktiviert [80, 81]. Sollte eine anschließende suffiziente Antikoagulation mit Heparin zwingend erforderlich sein (z. B. extrakorporale Membranoxygenierung, Operationen mit Einsatz der Herz-Lungen-Maschine, periprozedurale Heparinisierung im Herzkatheterlabor oder OP) muss die Gabe von AA besonders kritisch evaluiert werden. Bei diesen Patienten ist der Einsatz von PPSB wahrscheinlich sicherer [80, 81].

Antidot-Auswahl sollte auch nach der geplanten Intervention und Grunderkrankung ausgerichtet werden

Nach Reversierung von FXa-Hemmern mit AA konnte keine Normalisierung der RVV-Test-CT beobachtet werden. In Ex-vivo-Spiking-Versuchen konnte die RVV-Test-CT nicht zurück in den Referenzbereich gebracht werden [75]. Dies liegt mutmaßlich daran, dass AA chemisch dem humanen FXa sehr ähnlich ist und es zu einem kompetitiven Mechanismus kommt, der einer Normalisierung der CT im Wege steht [75]. Dies gilt auch für die Messung der Plasmakonzentration. In der EMA-Zulassung wird explizit darauf hingewiesen, dass nach Gabe von AA keine Plasmaspiegel gemessen werden sollten, sondern die Wirkung ausschließlich klinisch zu beurteilen ist [82].

Alternativ kann auch der Einsatz des CytoSorb-Adsorber® zur raschen Elimination von FXa-Inhibitoren erwogen werden [83, 84].

## Plättchenhemmer

Thrombozytenaggregationshemmer werden zur Primärprophylaxe bei koronarer Herzerkrankung, als Rezidiv- und Sekundärprophylaxe nach akutem Koronarsyndrom, nach Stent-Implantation und ischämischem Schlaganfall sowie zur Prävention von Gefäßverschlüssen bei peripherer arterieller Verschlusskrankheit eingesetzt. Das Risiko einer chirurgischen Blutung wird durch Aspirin oder Clopidogrel allein um etwa 20 % und durch eine duale Thrombozytenaggregationshemmung um 50 % erhöht [4].

### Therapie

#### Desmopressin (DDAVP)

DDAVP in einer Dosierung von 0,3–0,4 µg/kgKG führt zu einer verstärkten Freisetzung des vWF und von FVIII aus dem Endothel und den Lebersinusoiden. Die Aktivität des vWF steigt bis zum 4Fachen an und erreicht ein Wirkmaximum nach etwa 1 h. Daneben wurde auch eine verstärkte Freisetzung von Tissue Plasminogen Activator (t-PA) mit leicht gesteigerter fibrinolytischer Aktivität beschrieben [85]. Die Konzentrationssteigerung insbesondere der hochmolekularen vWF-Multimere kann die primäre Hämostase nach Einnahme von Acetylsalicylsäure verbessern. Für ADP-Hemmer, wie Clopidogrel oder Prasugrel, gibt es keine schlüssigen Daten, die eine verbesserte Thrombozytenfunktion nach Verabreichung von DDAVP nahelegen. Liegt bereits eine starke Aktivierung des Endothels vor, wie beispielsweise nach schwerem Trauma, kann kein zusätzlicher Effekt durch die DDAVP-Gabe erwartet werden. Barletta et al. konnten bei SHT-Patient:innen unter Aspirin oder ADP-Hemmern positive Effekte nur bei ASS zeigen und auch nur dann, wenn ASS vorher niedrig dosierter (< 80 mg/Tag) verabreicht wurde [86]. DDAVP kann Krampfanfälle auslösen und zu einer hyponatriämischen Hypervolämie führen [87].

#### Plättchenkonzentrate

Die Transfusion von Plättchenkonzentraten zur Verbesserung der primären Hämostase erscheint eine mögliche Therapieoption. Die publizierten Daten zur Effektivität einer Plättchentransfusion bei Blutungen unter Plättchenhemmer sind allerdings durchaus ernüchternd. Bei Patient:innen, die unter Plättchenhemmern spontane intrakranielle Blutungen erlitten und mit Thrombozytenkonzentraten behandelt wurden, war die Mortalität in der Therapiegruppe höher als in der Standard-of-care-Gruppe [88]. Auch bei SHT-Patient:innen unter Plättchenhemmern konnte durch die Transfusion von Plättchenkonzentraten keine relevante Verbesserung des Outcomes erreicht werden [89, 90].

#### Hämadsorption von Ticagrelor

In kleinen Fallserien konnte mithilfe des CytoSorb-Adsorber® auch eine rasche Elimination von Ticagrelor im Rahmen von herzchirurgischen Eingriffen erreicht werden [91]. In 2 prospektiven Studien (TISORB [Ticagrelor CytoSorb® Hemoadsorption; NCT04131959] und CyTation [Ticagrelor Removal Study Using CytoSorb® 300 mL Device During CPB in Patients Undergoing Emergent Cardiothoracic Surgery, NCT04625764]) wird die Wertigkeit dieser Eliminationsmethode bei kardiochirurgischen Eingriffen unter Ticagrelor nun weiteruntersucht.

## Konzept eines individualisierten Hämotherapie-Algorithmus basierend auf VET

**Figure 2:**
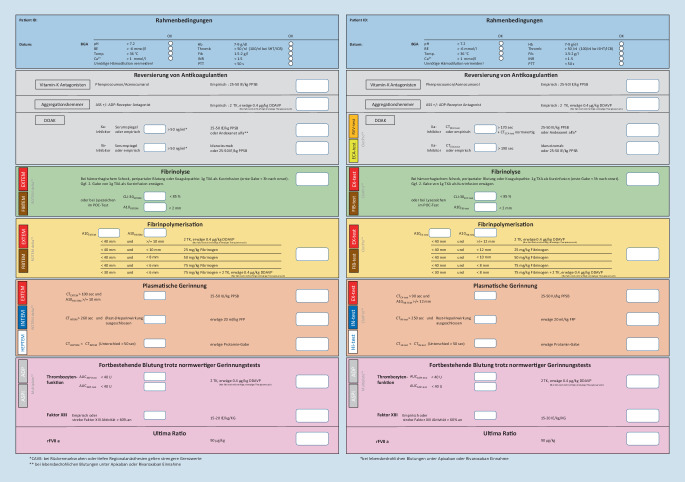

Im Folgenden werden die beiden Algorithmen für ROTEM® Delta (Abb. 2a) und ClotPro® (Abb. 2b) mit deren unterschiedlichen Grenzwerten vorgestellt.

### Rahmenbedingung

Bei der Behandlung einer akuten Hämorrhagie muss zunächst darauf geachtet werden, dass die notwendigen Rahmenbedingungen für eine suffiziente Hämostase erfüllt sind bzw. diese entsprechend optimiert werden. Zur Abschätzung einer bestehenden Acidose ist eine Blutgasanalyse (BGA) unumgänglich. In der Regel werden in der BGA auch der Kalziumspiegel und der Hämoglobinwert ausgewiesen. Eine Messung der aktuellen Körperkerntemperatur sollte jedenfalls regelmäßig erfolgen.

### Antikoagulanzien

Als weitere Ursache für eine akute Blutung, insbesondere bei älteren Patient:innen, ist auch eine Dauertherapie mit Antikoagulanzien nicht selten. Daher muss im Rahmen einer Hämorrhagie die gerinnungshemmende Wirkung des jeweiligen Präparates möglichst zeitnah antagonisiert werden. Die DOAC-spezifischen Antagonisten wurden bereits im Abschnitt „Antagonisierung von DOAC“ detailliert besprochen, dennoch soll auf einen Unterschied zwischen den beiden vorgestellten Algorithmen hingeweisen werden. Zur Detektion einer DOAC-Restwirkung stehen bei ClotPro® der RVV-Test für Xa-Inhibitoren und der ECA-Test für den Thrombininhibitor Dabigatran zur Verfügung (Grenzwerte: Tab. 4). Beim Einsatz eines ROTEM® Delta muss diese diagnostische Lücke durch entsprechende Labortests (DOAC-Plasma-Spiegel-Messungen, Messung der Anti-Xa-Aktivität) geschlossen werden.

### Lyse und Hyperfibinolyse

Die Wertigkeit spezifischer Lyse-Tests ist nicht hinlänglich geklärt. Eine bestehende Hyperfibrinolyse mit typischer Spindelbildung im EXTEM/EX-Test oder INTEM/IN-Test ist selten und besonders bei Traumapatient:innen mit hoher Mortalität assoziiert [92]. Eine „positive“ Kontrolle mittels APTEM/AP-Test scheint keinen weiteren diagnostischen Mehrwert zu bringen und sollte besonderen Indikation vorbehalten bleiben. Ein „0-Linien-FIBTEM/FIB-Test“ kann ebenfalls als Hinweis auf eine Hyperfibrinolyse gewertet werden [93]. Wichtig ist allerdings festzuhalten, dass fehlende Lyse-Zeichen in den viskoelastischen Standardtests eine Hyperfibrinolyse keinesfalls ausschließen [94]!

Hyperfibrinolysen können durch VET detektiert werden – negative VET-Tests schließen diese aber nicht sicher aus!

Die Indikation zur Verabreichung eines Antifibrinolytikums sollte besonders bei polytraumatisierten Patient:innen eher anhand der Schockschwere als auf Basis von VET-Ergebnissen erfolgen. Mittel der Wahl ist das Lysinanalogon Tranexamsäure in einer Dosierung von 15–20 mg/KG – in der Regel 1 g – über 10 min verabreicht, gefolgt von einem weiteren Gramm als prolongierte Gabe [10]. Eine Bolusgabe von TXA sollte vermieden werden, da dies zu Blutdruckabfällen führen kann [95].

### Gerinnselfestigkeit

Die Gerinnselfestigkeit (Clot-Amplitude, CA5/10) kann bereits nach 5 bzw. 10 min abgelesen werden und korreliert ausgezeichnet mit der endgültigen maximalen Gerinnselstärke [96]. Die maximale Clotstärke (MCF) von EXTEM/EX-Test oder INTEM/IN-Test ist ein Resultat aus der Interaktion von Thrombozyten, dem Fibrinnetzwerk und aktiviertem FXIII. Eine verminderte Gerinnselamplitude ist in hohem Maße mit Blutungen und Transfusionsbedarf assoziiert. Eine Gerinnselstärke in der A10 < 40 mm im EXTEM/EX-Test kann Hinweis auf einen erniedrigten Thrombozyten- und/oder Fibrinogenspiegel sein [97, 98]. Zur Differenzierung ist zusätzlich ein Fibrinpolymerisationstest im Sinne eines FIBTEM/FIB-Test notwendig.

#### Wertigkeit von FIBTEM/FIB-Test bei akuter Blutung

Fibrinogen (Gerinnungsfaktor I) spielt als Vorstufe des Fibrins eine essenzielle Rolle in der Gerinnselbildung. Physiologisch wird es in der Leber synthetisiert und liegt im Plasma in Konzentration von 2,0–4,0 g/l vor, kann aber als Akute-Phase-Protein in der Schwangerschaft oder bei Sepsis massiv ansteigen und Werte von > 6,0 g/l erreichen [99]. Daneben bindet Fibrinogen an den Glykoprotein-IIb/IIIa-Rezeptor von Thrombozyten und spielt daher auch in der primären Hämostase eine entscheidende Rolle [99–101].

Im Rahmen schwerer Blutverluste fällt der Fibrinogenspiegel früh und rasch ab. Niedrige Fibrinogenspiegel sind sowohl mit höherem Transfusionsbedarf als auch größerer Mortalität assoziiert [102, 103]. Die kritische Grenze, die mit erhöhter Blutungsneigung einhergeht, wird bei 1,5–2 g/l vermutet [102, 104]. Deshalb empfehlen aktuelle Guidelines eine frühzeitige Fibrinogensubstitution, spätestens ab Werten < 1,5 g/dl [10–12]. Mehrere Studien konnten hierbei eine gute Korrelation von ROTEM®-FIBTEM und Clotpro® FIB-Test und den nach der Clauss-Methode gemessenen Fibrinogenspiegeln bestätigen [44, 45, 97]. Eine verminderte Amplitude im FIBTEM/FIB-Test nach 10 min Laufzeit im ROTEM® < 10 mm und im ClotPro® < 12 mm gilt somit als Hinweis auf eine bestehende Hypofibrinogenämie und als Indikation für eine Fibrinogensubstitution [97, 105].

Nach Fibrinogenapplikation sollte die CA 10 im FIBTEM/FIB-Test zumindest einen Zielwert > 10 mm/> 12 mm erreichen. Dies kann in der Regel durch die Verabreichung von 25–50 mg/kgKG Fibrinogenkonzentrat erreicht werden. Im Zuge der frühen Schwerverletztenversorgung konnte gezeigt werden, dass die gezielte Gabe von Fibrinogenkonzentrat die Rate an Massivtransfusionen, im Vergleich zur alleinigen Verabreichung von FFP, signifikant reduzierte [24, 25, 106–108].

Jedes VET-Gerät hat unterschiedliche Mess- bzw. Grenzwerte, daher müssen auch Therapie-Algorithmen individuell an die Geräte angepasst werden!

#### Erniedrigte Clot-Amplitude im EXTEM/EX-Test oder INTEM/IN-Test bei normalem FIBTEM/FIB-Test

**Figure 3:**
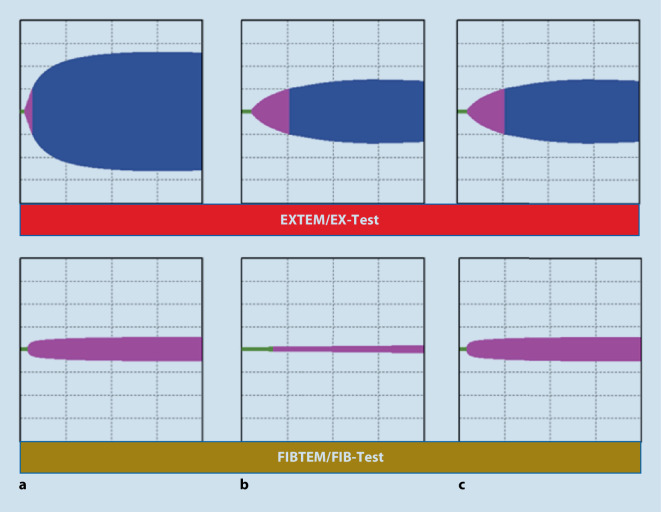

Eine reduzierte Clot-Amplitude (CA) nach 5 bzw. 10 min im EXTEM/EX-TEST, bei erhaltener Gerinnselamplitude im FIBTEM/FIB-Test, korrespondiert mit einer Thrombozytopenie. Erreicht der FIBTEM/FIB-Test den Grenzwert von > 10 mm/> 12 mm und ist die CA10 im EXTEM/EX-TEST weiter < 40 mm, kann dies ein Indiz für eine Thrombozytopenie sein und somit eine Indikation für die Verabreichung von Thrombozytenkonzentrate begründen. Die Autoren möchten an dieser Stelle aber hervorheben, dass nur Patienten mit klinischen Blutungszeichen einer Intervention bedürfen, und dass die hier aufgeführten Grenzwerte natürlich immer einer gewissen Schwankungsbreite unterliegen. Somit sollte am Ende immer die Klinik des Patienten über eine Therapie entscheiden (Abb. 3).

### Wertigkeit der Clotting time

Im nächsten Schritt erfolgt die viskoelastische Erfassung der Gerinnselbildungszeit. Die Clotting time (CT) ist zwar das erste verfügbare Messergebnis, wird aber nicht nur von der Aktivität der Gerinnungsfaktoren und von Antikoagulanzien, wie Heparin, VKA oder DOAC, beeinflusst, sondern auch in hohem Maß von der Konzentration an verfügbarem Fibrinogen [109]. Daher ist zur korrekten Einschätzung der CT die simultane Messung der Fibrinpolymerisation (FIBTEM/FIB-Test) erforderlich. Erst bei normaler FIBTEM/FIB-Test-Clot-Amplitude und unverändert verlängerter CT kann dies als Hinweis auf eine eingeschränkte Thrombingenerierung gewertet werden. Die Thrombinbildung kann mithilfe von PPSB oder Plasma augmentiert werden. Die übliche Dosis von PPSB liegt hier bei 25–50 U/kgKG. Da die Gerinnungsaktivität im Plasma relativ gering ist, müssen zur suffizienten Anhebung des Gerinnungspotenzials ausreichende Volumina (20–30 ml/kgKG) verabreicht werden [110].

Bei einer isolierten Verlängerung der CT im INTEM/IN-Test kann als Blutungsursache auch eine Restwirkung von unfraktioniertem Heparin vermutet werden. Zum Ausschluss einer Restheparinwirkung sollte daher, in dieser speziellen Konstellation, zusätzlich ein HEPTEM/HEP-Test durchgeführt werden. Verkürzt sich die CT im HEPTEM/HEP-Test, verglichen zum INTEM/IN-Test, kann dies als Hinweise auf eine bestehende Heparinwirkung interpretiert werden, die dann durch Protamin reversiert werden kann [47].

Die im initialen Algorithmus noch standardmäßig aufgeführte Plättchenfunktionsdiagnostik mit dem „Multiplate®“ wurde mangels valider Daten in dem vorgestellten Update aus der Routinediagnostik herausgenommen und wird nur noch bei fortbestehender Blutung trotz sonst normwertigen Gerinnungstests empfohlen. Darüber hinaus sollten bei persistierender Blutung trotz Algorithmus-konformer Hämotherapie unbedingt zwei weitere Limitationen der VET-Verfahren bedacht werden. Der Mangel an vWF und an FXIII kann durch VET-Methoden nicht erfasst werden und müsste ggf. empirisch therapiert werden. Die „Off-label“-Verabreichung von rekombinantem FVIIa (NovoSeven®) als Ultima-Ratio-Therapie sehen die Autoren kritisch, nicht zuletzt wegen fehlender Evidenzdaten, den hohen Kosten und dem gehäuften Auftreten thromboembolischer Ereignisse [111].

### Therapie nach dem Blutungsnotfall

Das Risiko, thromboembolische (TE) Komplikationen nach einem schweren Blutungsereignis zu entwickeln, ist auch bei frühem Einsatz medikamentöser Thromboseprophylaxe beispielsweise mit niedermolekularen Heparinen deutlich erhöht [112]. Die foudroyante Lungenembolie (LE) stellt hierbei eine häufige Todesursache nach schweren, traumaassoziierten Blutungen dar [113]. Eine frühe medikamentöse TE-Prophylaxe kann zu einer signifikanten Reduktion der Inzidenz tiefer Venenthrombosen (TVT) und von LE bei intensivmedizinischen und chirurgischen Patienten beitragen [114]. Niedermolekulare Heparine (NMH) führten zu einem geringeren Risiko von TVT und LE im Vergleich zu unfraktioniertem Heparin (UFH) und sollten deshalb bevorzugt eingesetzt werden [115, 116]. Eine erhöhte Rate an Blutungsereignissen unter Antikoagulation scheint hierbei zumindest in prophylaktischer Dosierung weder bei UFH noch NMH aufzutreten [117].

Die Gefahr von thromboembolischen Ereignissen ist ebenso groß, wie die Gefahr durch Blutungen!

## Evidenz

Ziel dieses aktualisierten Algorithmus ist, sowohl die Ergebnisse rezenter wissenschaftlicher Daten zu würdigen als auch der Verfügbarkeit neuer Reagenzien, welche die diagnostischen Möglichkeiten VET-Verfahren erweitern, Rechnung zu tragen.

Koagulopathien können vielfältige Ursachen haben, die durch Standardgerinnungstests oftmals unzureichend charakterisiert werden. Der Stellenwert von VET-Verfahren zur raschen Diagnostik einer zugrunde liegenden Koagulopathie wurde bislang in zahlreichen Studien sowohl aus dem Traumabereich als auch der Kardio- und Leberchirurgie oder im Rahmen von peripartalen Blutungen belegt [24, 26, 118, 119]. Dabei konnten zumeist eine signifikante Reduktion des Transfusionsbedarfs sowie in manchen Studien auch Überlebensvorteile gezeigt werden [22, 26, 120, 121].

Gonzales et al. konnten an 100 Schwerverletzten nachweisen, dass durch den Einsatz von VET (TEG 5000®) trotz geringerer Transfusion von Erythrozytenkonzentraten, FFP und Plättchenkonzentraten eine, wenn auch knapp signifikante Reduktion der Mortalität erreicht wurde [22]. In der bislang größten Studie an Traumapatient:innen (iTACTIC) konnten Baksaas-Aasen et al. keine signifikanten Überlebensvorteile durch die Anwendung von VET-Verfahren (ROTEM® oder TEG 5000®) im Vergleich zu SGT nachweisen. Nur in der vorher definierten Subgruppe von Patient:innen mit SHT zeigte sich eine signifikante Reduktion der Mortalität durch den Einsatz der VET-Methoden. Es ist allerdings kritisch anzumerken, dass bei iTACTIC, entsprechend der vorher festgelegten Definition, nur etwa ein Drittel der Patient:innen tatsächlich koagulopathisch war. Ein weiterer Kritikpunkt an der Studie ist, dass alle Patient:innen Blutprodukte in einem fixen 1:1:1-Verhältnis erhielten und somit keine individualisierte Gerinnungstherapie stattfand [105]. Auch in der RETIC-Studie von Innerhofer et al. konnten keine Überlebensvorteile durch den Einsatz von VET-Verfahren gezeigt werden. Die Rate an Massivtransfusionen war allerdings in der Kontrollgruppe signifikant höher [25].

Der in dieser Arbeit publizierte Hämotherapie-Algorithmus basiert auf ROTEM®-Delta- und ClotPro®-Messungen und ist nicht auf andere VET-Verfahren wie TEG6s®, TEG 5000® oder Quantra® übertragbar.

Selbst durch die Kombination viskoelastischer und aggregometrischer Verfahren können einige potenzielle Ursachen für perioperative Koagulopathien nicht diagnostiziert werden. So kann der Einfluss von niedermolekularen Heparinen, Faktor-Xa-Inhibitoren und DOAC, vWF und FXIII mit den gängigen Standardtests nicht mit hinreichender Sicherheit nachgewiesen werden.

Das Ziel der Expertenrunde war, unter Berücksichtigung einer evidenzbasierten Therapieeskalation einen problemlos in den klinischen Alltag integrierbaren POC-Algorithmus zu entwerfen. Praktikabilität und Wirksamkeit des Algorithmus wurden bislang nicht in prospektiven randomisierten Studien untersucht, sodass der Evidenzgrad des hier präsentierten Algorithmus lediglich als 4b (laut „Ärztliches Zentrum für Qualität in der Medizin“) einzustufen ist.

### Supplementary Information






## Anhang

### Wichtige Hinweise

Medizinische Erkenntnisse unterliegen einem fortgesetzten Wandel, der sowohl durch aktuelle Forschungsergebnisse als auch auf individuelle klinische Erfahrungen begründet wird. Der vorgelegte Therapie-Algorithmus wurde von den Autoren mit großer Sorgfalt erstellt, und die vorgestellten therapeutischen Angaben wurden hinsichtlich Indikation und Dosierung wurden dem aktuellen Wissenstand entsprechen dargestellt. Für den Nutzer des Algorithmus besteht allerdings trotzdem die Verpflichtung, die Hämotherapie in eigener Verantwortung durchzuführen. Der vorgestellte Algorithmus bezieht sich nur auf die Therapie von Gerinnungsstörungen, die im Rahmen schwerer Blutungen auftreten können. VET-Ergebnisse außerhalb der im Algorithmus angegebenen Grenzwerte sollten nur dann therapiert werden, wenn sie im klinischen Kontext zu einer Blutung stehen oder bei sehr hohem Blutungsrisiko. Durch die Richtlinien der Bundesärztekammer zur Qualitätssicherung quantitativer laboratoriumsmedizinischer Untersuchungen wurden verbindliche Anforderungen für regelmäßige interne Qualitätskontrollen, in der Regel mit Kontrollmaterialien des Herstellers, festgelegt, die unbedingt einzuhalten sind.

## References

[1] Wu WC, Smith TS, Henderson WG, Eaton CB, Poses RM, Uttley G, Mor V, Sharma SC, Vezeridis M, Khuri SF, Friedmann PD (2010). Operative blood loss, blood transfusion, and 30-day mortality in older patients after major noncardiac surgery. Ann Surg.

[2] Hamada SR, Garrigue D, Nougue H, Meyer A, Boutonnet M, Meaudre E, Culver A, Gaertner E, Audibert G, Vigué B, Duranteau J, Godier A (2022). Impact of platelet transfusion on outcomes in trauma patients. Crit Care.

[3] Rhoades R, French Z, Yang A, Walsh K, Drelich DA, McKenzie SE (2023). Perioperative outcomes of patients with bleeding disorders undergoing major surgery at an academic hemophilia treatment center. Clin Appl Thromb Hemost.

[4] Chassot PG, Delabays A, Spahn DR (2007). Perioperative use of anti-platelet drugs. Best Pract Res Clin Anaesthesiol.

[5] Hofer H, Oberladstätter D, Schlimp CJ, Voelckel W, Zipperle J, Lockie C, Grottke O, Osuchowski M, Schöchl H (2022) Role of DOAC plasma concentration on perioperative blood loss and transfusion requirements in patients with hip fractures. Eur J Trauma Emerg Surg 10.1007/s00068-022-02041-7 35841427

[6] Bruckbauer M, Prexl O, Voelckel W, Ziegler B, Grottke O, Maegele M, Schöchl H (2019). Impact of direct oral anticoagulants in patients with hip fractures. J Orthop Trauma.

[7] Prexl O, Bruckbauer M, Voelckel W, Grottke O, Ponschab M, Maegele M, Schöchl H (2018). The impact of direct oral anticoagulants in traumatic brain injury patients greater than 60-years-old. Scand J Trauma Resusc Emerg Med.

[8] Cap A, Hunt BJ (2015). The pathogenesis of traumatic coagulopathy. Anaesthesia.

[9] Haas T, Fries D, Tanaka KA, Asmis L, Curry NS, Schöchl H (2015). Usefulness of standard plasma coagulation tests in the management of perioperative coagulopathic bleeding: is there any evidence?. Br J Anaesth.

[10] Rossaint R, Afshari A, Bouillon B, Cerny V, Cimpoesu D, Curry N, Duranteau J, Filipescu D, Grottke O, Grønlykke L, Harrois A, Hunt BJ, Kaserer A, Komadina R, Madsen MH, Maegele M, Mora L, Riddez L, Romero CS, Samama CM, Vincent JL, Wiberg S, Spahn DR (2023). The European guideline on management of major bleeding and coagulopathy following trauma: sixth edition. Crit Care.

[11] Kietaibl S, Ahmed A, Afshari A, Albaladejo P, Aldecoa C, Barauskas G, De Robertis E, Faraoni D, Filipescu DC, Fries D, Godier A, Haas T, Jacob M, Lancé MD, Llau JV, Meier J, Molnar Z, Mora L, Rahe-Meyer N, Samama CM, Scarlatescu E, Schlimp C, Wikkelsø AJ, Zacharowski K (2023). Management of severe peri-operative bleeding: guidelines from the European society of anaesthesiology and intensive care: second update 2022. Eur J Anaesthesiol.

[12] Vlaar APJ, Dionne JC, de Bruin S, Wijnberge M, Raasveld SJ, van Baarle F, Antonelli M, Aubron C, Duranteau J, Juffermans NP, Meier J, Murphy GJ, Abbasciano R, Müller MCA, Lance M, Nielsen ND, Schöchl H, Hunt BJ, Cecconi M, Oczkowski S (2021). Transfusion strategies in bleeding critically ill adults: a clinical practice guideline from the European society of intensive care medicine. Intensive Care Med.

[13] Brustia R, Monsel A, Skurzak S, Schiffer E, Carrier FM, Patrono D, Kaba A, Detry O, Malbouisson L, Andraus W, Vandenbroucke-Menu F, Biancofiore G, Kaido T, Compagnon P, Uemoto S, Rodriguez Laiz G, De Boer M, Orloff S, Melgar P, Buis C, Zeillemaker-Hoekstra M, Usher H, Reyntjens K, Baird E, Demartines N, Wigmore S, Scatton O (2022). Guidelines for perioperative care for liver transplantation: enhanced recovery after surgery (ERAS) recommendations. Transplantation.

[14] Yoon U, Bartoszko J, Bezinover D, Biancofiore G, Forkin KT, Rahman S, Spiro M, Raptis DA, Kang Y (2022). Intraoperative transfusion management, antifibrinolytic therapy, coagulation monitoring and the impact on short-term outcomes after liver transplantation—a systematic review of the literature and expert panel recommendations. Clin Transplant.

[15] Boer C, Meesters MI, Milojevic M, Benedetto U, Bolliger D, von Heymann C, Jeppsson A, Koster A, Osnabrugge RL, Ranucci M, Ravn HB, Vonk ABA, Wahba A, Pagano D (2018). 2017 EACTS/EACTA guidelines on patient blood management for adult cardiac surgery. J Cardiothorac Vasc Anesth.

[16] Annecke T, Lier H, Girard T, Korte W, Pfanner G, Schlembach D, Tiebel O, von Heymann C (2022). Peripartum hemorrhage, diagnostics and treatment : update of the S2k guidelines AWMF 015/063 from august 2022. Anaesthesiologie.

[17] Stanworth SJ, Dowling K, Curry N, Doughty H, Hunt BJ, Fraser L, Narayan S, Smith J, Sullivan I, Green L (2022). Haematological management of major haemorrhage: a British society for haematology guideline. Br J Haematol.

[18] A.e. V., AWMF-Leitlinie 187-023: S3-Leitlinie Polytrauma/Schwerverletzten-Behandlung, Online (Stand 7. Mai 2023) (2022).

[19] Schlembach D, Mörtl MG, Girard T, Arzt W, Beinder E, Brezinka C, Chalubinski K, Fries D, Gogarten W, Hackelöer BJ, Helmer H, Henrich W, Hösli I, Husslein P, Kainer F, Lang U, Pfanner G, Rath W, Schleussner E, Steiner H, Surbek D, Zimmermann R (2014). Management of postpartum hemorrhage (PPH): algorithm of the interdisciplinary D-A-CH consensus group PPH (Germany—Austria—Switzerland). Anaesthesist.

[20] Bundesärztekammer, Querschnitts-Leitlinien zur Therapie mit Blutkomponenten und Plasmaderivaten Gesamtnovelle 2020, Online (Stand 7. Mai 2023) (2020).

[21] Gratz J, Güting H, Thorn S, Brazinova A, Görlinger K, Schäfer N, Schöchl H, Stanworth S, Maegele M (2019). Protocolised thromboelastometric-guided haemostatic management in patients with traumatic brain injury: a pilot study. Anaesthesia.

[22] Gonzalez E, Moore EE, Moore HB, Chapman MP, Chin TL, Ghasabyan A, Wohlauer MV, Barnett CC, Bensard DD, Biffl WL, Burlew CC, Johnson JL, Pieracci FM, Jurkovich GJ, Banerjee A, Silliman CC, Sauaia A (2016). Goal-directed hemostatic resuscitation of trauma-induced coagulopathy: a pragmatic randomized clinical trial comparing a viscoelastic assay to conventional coagulation assays. Ann Surg.

[23] Redfern RE, Fleming K, March RL, Bobulski N, Kuehne M, Chen JT, Moront M (2019). Thrombelastography-directed transfusion in cardiac surgery: impact on postoperative outcomes. Ann Thorac Surg.

[24] Schöchl H, Nienaber U, Maegele M, Hochleitner G, Primavesi F, Steitz B, Arndt C, Hanke A, Voelckel W, Solomon C (2011). Transfusion in trauma: thromboelastometry-guided coagulation factor concentrate-based therapy versus standard fresh frozen plasma-based therapy. Crit Care.

[25] Innerhofer P, Fries D, Mittermayr M, Innerhofer N, von Langen D, Hell T, Gruber G, Schmid S, Friesenecker B, Lorenz IH, Ströhle M, Rastner V, Trübsbach S, Raab H, Treml B, Wally D, Treichl B, Mayr A, Kranewitter C, Oswald E (2017). Reversal of trauma-induced coagulopathy using first-line coagulation factor concentrates or fresh frozen plasma (RETIC): a single-centre, parallel-group, open-label, randomised trial. Lancet Haematol.

[26] Weber CF, Görlinger K, Meininger D, Herrmann E, Bingold T, Moritz A, Cohn LH, Zacharowski K (2012). Point-of-care testing: a prospective, randomized clinical trial of efficacy in coagulopathic cardiac surgery patients. Anesthesiology.

[27] Ziegler B, Voelckel W, Zipperle J, Grottke O, Schöchl H (2019). Comparison between the new fully automated viscoelastic coagulation analysers TEG 6s and ROTEM Sigma in trauma patients: a prospective observational study. Eur J Anaesthesiol.

[28] Zlotnik D, Abdallah GA, Lang E, Boucebci KJ, Gautier CH, François A, Gaussem P, Godier A (2023) Assessment of a quantra-guided hemostatic algorithm in high-bleeding-risk cardiac surgery. J Cardiothorac Vasc Anesth 10.1053/j.jvca.2023.01.034 36822891

[29] Oberladstätter D, Voelckel W, Schlimp C, Zipperle J, Ziegler B, Grottke O, Schöchl H (2021). A prospective observational study of the rapid detection of clinically-relevant plasma direct oral anticoagulant levels following acute traumatic injury. Anaesthesia.

[30] Pommer P, Oberladstätter D, Schlimp CJ, Zipperle J, Voelckel W, Lockie C, Osuchowski M, Schöchl H (2022) Multiplate platelet function testing upon emergency room admission fails to provide useful information in major trauma patients not on platelet inhibitors. J Clin Med (11(9)) 10.3390/jcm11092578 PMC9100631 35566704

[31] Tantry US, Hartmann J, Neal MD, Schöechl H, Bliden KP, Agarwal S, Mason D, Dias JD, Mahla E, Gurbel PA (2022). The role of viscoelastic testing in assessing peri-interventional platelet function and coagulation. Platelets.

[32] Weber CF, Zacharowski K, Brün K, Volk T, Martin EO, Hofer S, Kreuer S (2013). Basic algorithm for point-of-care based hemotherapy: perioperative treatment of coagulopathic patients. Anaesthesist.

[33] Hartert H (1948). Blutgerinnungsstudien mit der Thrombelastographie, einem neuen Untersuchungsverfahren. Klin Wochenschr.

[34] Hagemo JS, Næss PA, Johansson P, Windeløv NA, Cohen MJ, Røislien J, Brohi K, Heier HE, Hestnes M, Gaarder C (2013). Evaluation of TEG(®) and RoTEM(®) inter-changeability in trauma patients. Injury.

[35] Dias JD, Haney EI, Mathew BA, Lopez-Espina CG, Orr AW, Popovsky MA (2017). New-generation thromboelastography: comprehensive evaluation of citrated and heparinized blood sample storage effect on clot-forming variables. Arch Pathol Lab Med.

[36] Michelson EA, Cripps MW, Ray B, Winegar DA, Viola F (2020). Initial clinical experience with the quantra qStat system in adult trauma patients. Trauma Surg Acute Care Open.

[37] Mann KG, Brummel K, Butenas S (2003). What is all that thrombin for?. J Thromb Haemost.

[38] Hoffman M, Monroe DM (2001). A cell-based model of hemostasis. Thromb Haemost.

[39] Chee YL, Crawford JC, Watson HG, Greaves M (2008). Guidelines on the assessment of bleeding risk prior to surgery or invasive procedures. British committee for standards in haematology. Br J Haematol.

[40] Levy JH, Szlam F, Wolberg AS, Winkler A (2014). Clinical use of the activated partial thromboplastin time and prothrombin time for screening: a review of the literature and current guidelines for testing. Clin Lab Med.

[41] Schöchl H, Voelckel W, Grassetto A, Schlimp CJ (2013). Practical application of point-of-care coagulation testing to guide treatment decisions in trauma. J Trauma Acute Care Surg.

[42] Neal MD, Moore EE, Walsh M, Thomas S, Callcut RA, Kornblith LZ, Schreiber M, Ekeh AP, Singer AJ, Lottenberg L, Foreman M, Evans S, Winfield RD, Goodman MD, Freeman C, Milia D, Saillant N, Hartmann J, Achneck HE (2020). A comparison between the TEG 6s and TEG 5000 analyzers to assess coagulation in trauma patients. J Trauma Acute Care Surg.

[43] Laukova K, Petrikova V, Poloniova L, Babulicova L, Wsolova L, Haas T (2023). Determination of reference ranges for the Clotpro® thromboelastometry device in paediatric patients. Br J Anaesth.

[44] Infanger L, Dibiasi C, Schaden E, Ulbing S, Wiegele M, Lacom C, Gratz J (2021). Comparison of the new viscoelastic coagulation analyzer ClotPro® With ROTEM® Delta and conventional coagulation tests in critically Ill patients with COVID-19. Front Med (lausanne).

[45] Yoshii R, Sawa T, Kawajiri H, Amaya F, Tanaka KA, Ogawa S (2022). A comparison of the ClotPro system with rotational thromboelastometry in cardiac surgery: a prospective observational study. Sci Rep.

[46] Kvint S, Gutierrez A, Venezia A, Maloney E, Schuster J, Kumar MA (2022). Application of a TEG-Platelet mapping algorithm to guide reversal of antiplatelet agents in adults with mild-to-moderate traumatic brain injury: an observational pilot study. Neurocrit Care.

[47] Sahli SD, Castellucci C, Roche TR, Rössler J, Spahn DR, Kaserer A (2022). The impact of direct oral anticoagulants on viscoelastic testing—a systematic review. Front Cardiovasc Med.

[48] Korpallová B, Samoš M, Bolek T, Kühnelová L, Škorňová I, Kubisz P, Staško J, Mokáň M (2021). ROTEM testing for direct oral anticoagulants. Semin Thromb Hemost.

[49] Mohr J, Ruchholtz S, Hildebrand F, Flohé S, Frink M, Witte I, Weuster M, Fröhlich M, van Griensven M, Keibl C, Mommsen P (2013). Induced hypothermia does not impair coagulation system in a swine multiple trauma model. J Trauma Acute Care Surg.

[50] George MJ, Burchfield J, MacFarlane B, Wang YW, Cardenas JC, White NJ, Gill BS, Wade CE (2018). Multiplate and TEG platelet mapping in a population of severely injured trauma patients. Transfus Med.

[51] Schriner JB, George MJ, Cardenas JC, Olson SD, Mankiewicz KA, Cox CS, Gill BS, Wade CE (2022). Platelet function In trauma: is current technology in function testing missing the mark in injured patients?. Shock.

[52] Bolliger D, Lancé MD, Siegemund M (2021). Point-of-Care platelet function monitoring: implications for patients with platelet inhibitors in cardiac surgery. J Cardiothorac Vasc Anesth.

[53] Martini WZ, Pusateri AE, Uscilowicz JM, Delgado AV, Holcomb JB (2005). Independent contributions of hypothermia and acidosis to coagulopathy in swine. J Trauma.

[54] Martini WZ, Holcomb JB (2007). Acidosis and coagulopathy: the differential effects on fibrinogen synthesis and breakdown in pigs. Ann Surg.

[55] Darlington DN, Kheirabadi BS, Delgado AV, Scherer MR, Martini WZ, Dubick MA (2011). Coagulation changes to systemic acidosis and bicarbonate correction in swine. J Trauma.

[56] de Vrij EL, Vogelaar PC, Goris M, Houwertjes MC, Herwig A, Dugbartey GJ, Boerema AS, Strijkstra AM, Bouma HR, Henning RH (2014). Platelet dynamics during natural and pharmacologically induced torpor and forced hypothermia. Plos One.

[57] Wolberg AS, Meng ZH, Monroe DM, Hoffman M (2004). A systematic evaluation of the effect of temperature on coagulation enzyme activity and platelet function. J Trauma.

[58] Van Poucke S, Stevens K, Marcus AE, Hypothermia ML (2014). effects on platelet function and hemostasis. Thromb J.

[59] Varga-Szabo D, Braun A, Nieswandt B (2009). Calcium signaling in platelets. J Thromb Haemost.

[60] Kronstedt S, Roberts N, Ditzel R, Elder J, Steen A, Thompson K, Anderson J, Siegler J (2022). Hypocalcemia as a predictor of mortality and transfusion. A scoping review of hypocalcemia in trauma and hemostatic resuscitation. Transfusion.

[61] James MF, Roche AM (2004). Dose-response relationship between plasma ionized calcium concentration and thrombelastography. J Cardiothorac Vasc Anesth.

[62] Lehmann M, Wallbank AM, Dennis KA, Wufsus AR, Davis KM, Rana K, Neeves KB (2015). On-chip recalcification of citrated whole blood using a microfluidic herringbone mixer. Biomicrofluidics.

[63] Uijttewaal WS, Nijhof EJ, Bronkhorst PJ, Den Hartog E, Heethaar RM (1993). Near-wall excess of platelets induced by lateral migration of erythrocytes in flowing blood. Am J Physiol.

[64] Altiok E, Marx N (2018). Oral anticoagulation. Dtsch Ärztebl Int.

[65] Sahai T, Tavares MF, Sweeney JD (2017). Rapid response to intravenous vitamin K may obviate the need to transfuse prothrombin complex concentrates. Transfusion.

[66] Grottke O, Rossaint R, Henskens Y, van Oerle R, Ten HC, Spronk HM (2013). Thrombin generation capacity of prothrombin complex concentrate in an in vitro dilutional model. Plos One.

[67] Goldstein JN, Refaai MA, Milling TJ, Lewis B, Goldberg-Alberts R, Hug BA, Sarode R (2015). Four-factor prothrombin complex concentrate versus plasma for rapid vitamin K antagonist reversal in patients needing urgent surgical or invasive interventions: a phase 3b, open-label, non-inferiority, randomised trial. Lancet.

[68] Faulkner H, Chakankar S, Mammi M, Lo JYT, Doucette J, Al-Otaibi N, Abboud J, Le A, Mekary RA, Bunevicius A (2021). Safety and efficacy of prothrombin complex concentrate (PCC) for anticoagulation reversal in patients undergoing urgent neurosurgical procedures: a systematic review and metaanalysis. Neurosurg Rev.

[69] Lip GYH, Keshishian A, Li X, Hamilton M, Masseria C, Gupta K, Luo X, Mardekian J, Friend K, Nadkarni A, Pan X, Baser O, Deitelzweig S (2018). Effectiveness and safety of oral anticoagulants among nonvalvular atrial fibrillation patients. Stroke.

[70] Douxfils J, Adcock DM, Bates SM, Favaloro EJ, Gouin-Thibault I, Guillermo C, Kawai Y, Lindhoff-Last E, Kitchen S, Gosselin RC (2021). 2021 Update of the international council for standardization in haematology recommendations for laboratory measurement of direct oral anticoagulants. Thromb Haemost.

[71] Poulsen BK, Grove EL, Husted SE (2012). New oral anticoagulants: a review of the literature with particular emphasis on patients with impaired renal function. Drugs.

[72] Douxfils J, Ageno W, Samama CM, Lessire S, Ten HC, Verhamme P, Dogné JM, Mullier F (2018). Laboratory testing in patients treated with direct oral anticoagulants: a practical guide for clinicians. J Thromb Haemost.

[73] Connolly SJ, Crowther M, Eikelboom JW, Gibson CM, Curnutte JT, Lawrence JH, Yue P, Bronson MD, Lu G, Conley PB, Verhamme P, Schmidt J, Middeldorp S, Cohen AT, Beyer-Westendorf J, Albaladejo P, Lopez-Sendon J, Demchuk AM, Pallin DJ, Concha M, Goodman S, Leeds J, Souza S, Siegal DM, Zotova E, Meeks B, Ahmad S, Nakamya J, Milling TJ (2019). Full study report of andexanet alfa for bleeding associated with factor Xa inhibitors. N Engl J Med.

[74] Pollack CV, Reilly PA, van Ryn J, Eikelboom JW, Glund S, Bernstein RA, Dubiel R, Huisman MV, Hylek EM, Kam CW, Kamphuisen PW, Kreuzer J, Levy JH, Royle G, Sellke FW, Stangier J, Steiner T, Verhamme P, Wang B, Young L, Weitz JI (2017). Idarucizumab for dabigatran reversal—full cohort analysis. N Engl J Med.

[75] Oberladstätter D, Schlimp CJ, Zipperle J, Osuchowski MF, Voelckel W, Grottke O, Schöchl H (2021) Impact of idarucizumab and andexanet alfa on DOAC plasma concentration and ClotPro(®) clotting time: an Ex Vivo spiking study in a cohort of trauma patients. J Clin Med (10(16)) 10.3390/jcm10163476 PMC8396852 34441771

[76] Oberladstätter D, Voelckel W, Bruckbauer M, Zipperle J, Grottke O, Ziegler B, Schöchl H (2021). Idarucizumab in major trauma patients: a single centre real life experience. Eur J Trauma Emerg Surg.

[77] Simon A, Domanovits H, Ay C, Sengoelge G, Levy JH, Spiel AO (2017). The recommended dose of idarucizumab may not always be sufficient for sustained reversal of dabigatran. J Thromb Haemost.

[78] Hegemann I, Ganter C, Widmer CC, Becker M, Müller D, Spahn DR (2018). Ongoing redistribution of dabigatran necessitates repetitive application of idarucizumab. Br J Anaesth.

[79] Lu G, DeGuzman FR, Hollenbach SJ, Karbarz MJ, Abe K, Lee G, Luan P, Hutchaleelaha A, Inagaki M, Conley PB, Phillips DR, Sinha U (2013). A specific antidote for reversal of anticoagulation by direct and indirect inhibitors of coagulation factor Xa. Nat Med.

[80] Eche IM, Elsamadisi P, Wex N, Wyers MC, Brat GA, Cunningham K, Bauer KA (2019). Intraoperative unfractionated heparin unresponsiveness during endovascular repair of a ruptured abdominal aortic aneurysm following administration of andexanet alfa for the reversal of rivaroxaban. Pharmacotherapy.

[81] Watson CJ, Zettervall SL, Hall MM, Ganetsky M (2019). Difficult intraoperative heparinization following andexanet alfa administration. Clin Pract Cases Emerg Med.

[82] E.M. Agency, PRAC recommendations on signals Adopted at the 14–17 April 2020 PRAC meeting, Online (Stand 7. Mai 2023) (2020).

[83] Mair H, Jilek C, Haas B, Lamm P (2020). Ticagrelor and rivaroxaban elimination with cytosorb adsorber before urgent off-pump coronary bypass. Ann Thorac Surg.

[84] Buonocore M, Rex S, Degezelle K, Meyns B (2022). CytoSorb haemoadsorption for removal of apixaban—a proof-of-concept pilot case for a randomized controlled trial. J Clin Pharm Ther.

[85] Mannucci PM, Levi M (2007). Prevention and treatment of major blood loss. N Engl J Med.

[86] Barletta JF, Abdul-Rahman D, Hall ST, Mangram AJ, Dzandu JK, Frontera JA, Zach V (2020). The role of desmopressin on hematoma expansion in patients with mild traumatic brain injury prescribed pre-injury antiplatelet medications. Neurocrit Care.

[87] Wang Q, Alshayyah R, Yang B (2022). The efficacy and safety of desmopressin acetate applied for nocturia in benign prostatic hyperplasia patients: a systematic review and meta-analysis. Low Urin Tract Symptoms.

[88] Baharoglu MI, Cordonnier C, Al-Shahi RS, de Gans K, Koopman MM, Brand A, Majoie CB, Beenen LF, Marquering HA, Vermeulen M, Nederkoorn PJ, de Haan RJ, Roos YB (2016). Platelet transfusion versus standard care after acute stroke due to spontaneous cerebral haemorrhage associated with antiplatelet therapy (PATCH): a randomised, open-label, phase 3 trial. Lancet.

[89] Wolff C, Muakkassa F, Marley R, El-Khatib A, Docherty C, Muakkassa L, Stephen H, Salvator A (2022). Routine platelet transfusion in patients with traumatic intracranial hemorrhage taking antiplatelet medication: is it warranted?. Can J Surg.

[90] Thorn S, Güting H, Mathes T, Schäfer N, Maegele M (2019). The effect of platelet transfusion in patients with traumatic brain injury and concomitant antiplatelet use: a systematic review and meta-analysis. Transfusion.

[91] Harky A, Badran A (2021). Reducing antithrombotic-related bleeding risk in urgent and emergency cardiac surgery. Br J Cardiol.

[92] Schöchl H, Frietsch T, Pavelka M, Jámbor C (2009). Hyperfibrinolysis after major trauma: differential diagnosis of lysis patterns and prognostic value of thrombelastometry. J Trauma.

[93] Wang IJ, Park SW, Bae BK, Lee SH, Choi HJ, Park SJ, Ahn TY, Goh TS, Lee MJ, Yeom SR (2020). FIBTEM improves the sensitivity of hyperfibrinolysis detection in severe trauma patients: a retrospective study using thromboelastometry. Sci Rep.

[94] Raza I, Davenport R, Rourke C, Platton S, Manson J, Spoors C, Khan S, De’Ath HD, Allard S, Hart DP, Pasi KJ, Hunt BJ, Stanworth S, MacCallum PK, Brohi K (2013). The incidence and magnitude of fibrinolytic activation in trauma patients. J Thromb Haemost.

[95] Murao S, Nakata H, Roberts I, Yamakawa K (2021). Effect of tranexamic acid on thrombotic events and seizures in bleeding patients: a systematic review and meta-analysis. Crit Care.

[96] Meyer AS, Meyer MA, Sørensen AM, Rasmussen LS, Hansen MB, Holcomb JB, Cotton BA, Wade CE, Ostrowski SR, Johansson PI (2014). Thrombelastography and rotational thromboelastometry early amplitudes in 182 trauma patients with clinical suspicion of severe injury. J Trauma Acute Care Surg.

[97] Schöchl H, Cotton B, Inaba K, Nienaber U, Fischer H, Voelckel W, Solomon C (2011). FIBTEM provides early prediction of massive transfusion in trauma. Crit Care.

[98] Davenport R, Manson J, De’Ath H, Platton S, Coates A, Allard S, Hart D, Pearse R, Pasi KJ, MacCallum P, Stanworth S, Brohi K (2011). Functional definition and characterization of acute traumatic coagulopathy. Crit Care Med.

[99] Mosesson MW (2005). Fibrinogen and fibrin structure and functions. J Thromb Haemost.

[100] Schlimp CJ, Schöchl H (2014). The role of fibrinogen in trauma-induced coagulopathy. Hamostaseologie.

[101] Litvinov RI, Pieters M, de Lange-Loots Z, Weisel JW (2021). Fibrinogen and fibrin. Subcell Biochem.

[102] McQuilten ZK, Wood EM, Bailey M, Cameron PA, Cooper DJ (2017). Fibrinogen is an independent predictor of mortality in major trauma patients: a five-year statewide cohort study. Injury.

[103] Curry N, Rourke C, Davenport R, Beer S, Pankhurst L, Deary A, Thomas H, Llewelyn C, Green L, Doughty H, Nordmann G, Brohi K, Stanworth S (2015). Early cryoprecipitate for major haemorrhage in trauma: a randomised controlled feasibility trial. Br J Anaesth.

[104] Hagemo JS, Stanworth S, Juffermans NP, Brohi K, Cohen M, Johansson PI, Røislien J, Eken T, Næss PA, Gaarder C (2014). Prevalence, predictors and outcome of hypofibrinogenaemia in trauma: a multicentre observational study. Crit Care.

[105] Baksaas-Aasen K, Van Dieren S, Balvers K, Juffermans NP, Næss PA, Rourke C, Eaglestone S, Ostrowski SR, Stensballe J, Stanworth S, Maegele M, Goslings JC, Johansson PI, Brohi K, Gaarder C (2019). Data-driven development of ROTEM and TEG algorithms for the management of trauma hemorrhage: a prospective observational multicenter study. Ann Surg.

[106] Akbari E, Safari S, Hatamabadi H (2018). The effect of fibrinogen concentrate and fresh frozen plasma on the outcome of patients with acute traumatic coagulopathy: a quasi-experimental study. Am J Emerg Med.

[107] Kaserer A, Casutt M, Sprengel K, Seifert B, Spahn DR, Stein P (2018). Comparison of two different coagulation algorithms on the use of allogenic blood products and coagulation factors in severely injured trauma patients: a retrospective, multicentre, observational study. Scand J Trauma Resusc Emerg Med.

[108] Winearls J, Wullschleger M, Wake E, Hurn C, Furyk J, Ryan G, Trout M, Walsham J, Holley A, Cohen J, Shuttleworth M, Dyer W, Keijzers G, Fraser JF, Presneill J, Campbell D (2017). Fibrinogen early in severe trauma studY (FEISTY): study protocol for a randomised controlled trial. Trials.

[109] Gratz J, Schlimp CJ, Honickel M, Hochhausen N, Schöchl H, Grottke O (2020) Sufficient thrombin generation despite 95 % hemodilution: an in vitro experimental study. J Clin Med (9(12)) 10.3390/jcm9123805 PMC7760770 33255530

[110] Chowdary P, Tang A, Watson D, Besser M, Collins P, Creagh MD, Qureshi H, Rokicka M, Nokes T, Diprose P, Gill R (2018). Retrospective review of a prothrombin complex concentrate (Beriplex P/N) for the management of perioperative bleeding unrelated to oral anticoagulation. Clin Appl Thromb Hemost.

[111] Sozio MS, Chalasani N (2014). Activated recombinant factor VIIa should not be used in patients with refractory variceal bleeding: it is mostly ineffective, is expensive, and may rarely cause serious adverse events. Hepatology.

[112] Hamada SR, Espina C, Guedj T, Buaron R, Harrois A, Figueiredo S, Duranteau J (2017). High level of venous thromboembolism in critically ill trauma patients despite early and well-driven thromboprophylaxis protocol. Ann Intensive Care.

[113] Geerts WH, Code KI, Jay RM, Chen E, Szalai JP (1994). A prospective study of venous thromboembolism after major trauma. N Engl J Med.

[114] Alhazzani W, Lim W, Jaeschke RZ, Murad MH, Cade J, Cook DJ (2013). Heparin thromboprophylaxis in medical-surgical critically ill patients: a systematic review and meta-analysis of randomized trials. Crit Care Med.

[115] Beitland S, Sandven I, Kjærvik LK, Sandset PM, Sunde K, Eken T (2015). Thromboprophylaxis with low molecular weight heparin versus unfractionated heparin in intensive care patients: a systematic review with meta-analysis and trial sequential analysis. Intensive Care Med.

[116] Duranteau J, Taccone FS, Verhamme P, Ageno W (2018). European guidelines on perioperative venous thromboembolism prophylaxis: intensive care. Eur J Anaesthesiol.

[117] Ammane S, Glauser F, Robert-Ebadi H, Righini M, Blondon M (2022). Hospital mechanical thromboprophylaxis. Rev Med Suisse.

[118] Collins P (2022). Point-of-care coagulation testing for postpartum haemorrhage. Best Pract Res Clin Anaesthesiol.

[119] Lawson PJ, Moore HB, Moore EE, Stettler GR, Pshak TJ, Kam I, Silliman CC, Nydam TL (2017). Preoperative thrombelastography maximum amplitude predicts massive transfusion in liver transplantation. J Surg Res.

[120] Al Moosawi M, Trudeau J, Smith T, Lefebvre A, Shih AW (2021). ROTEM in the setting of liver transplant surgery reduces frozen plasma transfusion. Transfus Apher Sci.

[121] Jokinen S, Kuitunen A, Uotila J, Yli-Hankala A (2023). Thromboelastometry-guided treatment algorithm in postpartum haemorrhage: a randomised, controlled pilot trial. Br J Anaesth.

