# Supplementary material for: Update on point-of-care-based coagulation treatment: Systems, reagents, device-specific treatment algorithms
Source: Anaesthesiologie. 2024 Jan 23;73(2):110–23. [Article in German] doi: 10.1007/s00101-023-01368-z (PMC10850202; doi:10.1007/s00101-023-01368-z)
Supplement: Supplementary file 1 [file 101_2023_1368_MOESM1_ESM.pdf]

Algorithmus ROTEM delta
zur Gerinnungstherapie bei perioperativen Blutungen

Patient ID:

Rahmenbedingungen

Datum:

BGA

		OK			OK
pH	> 7.2	◯	Hb	7-9 g/dl	◯
BE	> -6 mmol/l	◯	Thromb	> 50 /nl (100/nl bei SHT/ICB)	◯
Temp.	> 36 °C	◯	Fib	1.5-2 g/l	◯
Ca^{2+}	> 1 mmol/l	◯	INR	< 1.5	◯
Unnötige Hämodilution vermeiden!			PTT	< 50 s	◯

Reversierung von Antikoagulantien

Vitamin-K Antagonisten – Phenprocoumon/Acenocumarol – Empirisch : 25-50 IE/kg PPSB

Aggregationshemmer – ASS +/- ADP-Rezeptor Antagonist – Empirisch : 2 TK, erwäge 0.4 µg/kg DDAVP (Nur falls noch nicht erfolgt; einmaliger Therapieversuch)

DOAK

Xa-Inhibitor	Serumspiegel oder empirisch	☐	> 50 ng/ml*	25-50 IE/kg PPSB oder Andexanet alfa**
IIa-Inhibitor	Serumspiegel oder empirisch	☐	> 50 ng/ml*	Idarucizumab oder 25-50 IE/kg PPSB

Fibrinolyse

EXTEM | FIBTEM | ROTEM delta™

Bei hämorrhagischem Schock, peripartaler Blutung oder Koagulopathie: 1g TXA als Kurzinfusion (erste Gabe < 3h nach onset). Ggf. 2. Gabe von 1g TXA als Kurzinfusion erwägen.

oder bei Lysezeichen im POC-Test

- $CLI\text{-}30_{EXTEM}$ ☐ < 85 %
- $A10_{FIBTEM}$ ☐ < 2 mm

Fibrinpolymerisation

EXTEM | FIBTEM | ROTEM delta™

$A10_{EXTEM}$		$A10_{FIBTEM}$	
< 40 mm	und	>/= 10 mm	2 TK, erwäge 0.4 µg/kg DDAVP (Nur falls noch nicht erfolgt; einmaliger Therapieversuch)
< 40 mm	und	< 10 mm	25 mg/kg Fibrinogen
< 40 mm	und	< 8 mm	50 mg/kg Fibrinogen
< 40 mm	und	< 6 mm	75 mg/kg Fibrinogen
< 30 mm	und	< 6 mm	75 mg/kg Fibrinogen + 2 TK, erwäge 0.4 µg/kg DDAVP (Nur falls noch nicht erfolgt; einmaliger Therapieversuch)

Plasmatische Gerinnung

EXTEM | INTEM | HEPTEM | ROTEM delta™

CT_{EXTEM} > 100 sec und $A10_{FIB\text{-}TEM}$ >/= 10 mm	☐	25-50 IU/kg PPSB
CT_{INTEM} > 260 sec und (Rest-)Heparinwirkung ausgeschlossen	☐	erwäge 20 ml/kg FFP
CT_{HEPTEM} < CT_{INTEM} (Unterschied > 50 sec)	☐	erwäge Protamin-Gabe

Fortbestehende Blutung trotz normwertiger Gerinnungstests

ADP | ASPI | Multiplate™

Thrombozyten-funktion

- $AUC_{ASPI\text{-}test}$ < 40 U ☐
- $AUC_{ADP\text{-}test}$ < 40 U ☐

2 TK, erwäge 0.4 µg/kg DDAVP (Nur falls noch nicht erfolgt; einmaliger Therapieversuch)

Faktor XIII – Empirisch oder strebe Faktor XIII Aktivität > 60% an ☐ – 15-20 IE/kg/KG

Ultima Ratio

rFVII a – 90 µg/kg

*CAVE: bei Rückenmarksnahen oder tiefen Regionalanästhesien gelten strengere Grenzwerte

** bei lebensbedrohlichen Blutungen unter Apixaban oder Rivaroxaban Einnahme
