# Supplementary material for: Update on point-of-care-based coagulation treatment: Systems, reagents, device-specific treatment algorithms
Source: Anaesthesiologie. 2024 Jan 23;73(2):110–23. [Article in German] doi: 10.1007/s00101-023-01368-z (PMC10850202; doi:10.1007/s00101-023-01368-z)
Supplement: Supplementary file 2 [file 101_2023_1368_MOESM2_ESM.pdf]

# Algorithmus ClotPro
## zur Gerinnungstherapie bei perioperativen Blutungen

**Patient ID:**

### Rahmenbedingungen

**Datum:**

**BGA**

| | | OK | | | OK |
|---|---|---|---|---|---|
| pH | > 7.2 | ◯ | Hb | 7-9 g/dl | ◯ |
| BE | > -6 mmol/l | ◯ | Thromb | > 50 /nl (100/nl bei SHT/ICB) | ◯ |
| Temp. | > 36 °C | ◯ | Fib | 1.5-2 g/l | ◯ |
| $Ca^{2+}$ | > 1 mmol/l | ◯ | INR | < 1.5 | ◯ |
| Unnötige Hämodilution vermeiden! | | | PTT | < 50 s | ◯ |

### Reversierung von Antikoagulantien

**Vitamin-K Antagonisten** — Phenprocoumon/Acenocumarol — Empirisch : 25-50 IE/kg PPSB

**Aggregationshemmer** — ASS +/- ADP-Rezeptor Antagonist — Empirisch : 2 TK, erwäge 0.4 µg/kg DDAVP (Nur falls noch nicht erfolgt; einmaliger Therapieversuch )

**DOAK** (ClotPro™: RVV-test, ECA-test)

| | | | | |
|---|---|---|---|---|
| Xa-Inhibitor | $CT_{RVV\text{-}test}$ oder empirisch | > 170 sec + $CT_{ECA\text{-}test}$ normwertig | 25-50 IE/kg PPSB oder Andexanet alfa* |
| IIa-Inhibitor | $CT_{ECA\text{-}test}$ oder empirisch | > 190 sec | Idarucizumab oder 25-50 IE/kg PPSB |

### Fibrinolyse

ClotPro™: EX-test, FIB-test

Bei hämorrhagischem Schock, peripartaler Blutung oder Koagulopathie: 1g TXA als Kurzinfusion (erste Gabe < 3h nach onset). Ggf. 2. Gabe von 1g TXA als Kurzinfusion erwägen.

oder bei Lysezeichen im POC-Test

- $CLI\text{-}30_{EXT\text{-}test}$ < 85 %
- $A10_{FIB\text{-}test}$ < 2 mm

### Fibrinpolymerisation

ClotPro™: EX-test, FIB-test

| $A10_{EX\text{-}test}$ | | $A10_{FIB\text{-}test}$ | |
|---|---|---|---|
| < 40 mm | und | >/= 12 mm | 2 TK, erwäge 0.4 µg/kg DDAVP (Nur falls noch nicht erfolgt; einmaliger Therapieversuch) |
| < 40 mm | und | < 12 mm | 25 mg/kg Fibrinogen |
| < 40 mm | und | < 10 mm | 50 mg/kg Fibrinogen |
| < 40 mm | und | < 8 mm | 75 mg/kg Fibrinogen |
| < 30 mm | und | < 8 mm | 75 mg/kg Fibrinogen + 2 TK, erwäge 0.4 µg/kg DDAVP (Nur falls noch nicht erfolgt; einmaliger Therapieversuch) |

### Plasmatische Gerinnung

ClotPro™: EX-test, IN-test, HI-test

| | |
|---|---|
| $CT_{EX\text{-}test}$ > 90 sec und $A10_{FIB\text{-}TEM}$ >/= 12 mm | 25-50 IU/kg PPSB |
| $CT_{IN\text{-}test}$ > 250 sec und Rest-Heparinwirkung ausgeschlossen | erwäge 20 ml/kg FFP |
| $CT_{HI\text{-}test}$ < $CT_{IN\text{-}test}$ (Unterschied > 50 sec) | erwäge Protamin-Gabe |

### Fortbestehende Blutung trotz normwertiger Gerinnungstests

Multiplate™: ADP, ASPI

**Thrombozyten-funktion**
- $AUC_{ASPI\text{-}test}$ < 40 U
- $AUC_{ADP\text{-}test}$ < 40 U

2 TK, erwäge 0.4 µg/kg DDAVP (Nur falls noch nicht erfolgt; einmaliger Therapieversuch)

**Faktor XIII** — Empirisch oder strebe Faktor XIII Aktivität > 60% an — 15-20 IE/kg/KG

### Ultima Ratio

**rFVII a** — 90 µg/kg

*bei lebensbedrohlichen Blutungen unter Apixaban oder Rivaroxaban Einnahme
